# Nutritional Deficiencies 3 Years After Sleeve Gastrectomy Can Be Limited by a Specialized Multivitamin Supplement

**DOI:** 10.1007/s11695-022-06256-w

**Published:** 2022-08-26

**Authors:** Laura Heusschen, Agnes A. M. Berendsen, Laura N. Deden, Eric J. Hazebroek, Edo O. Aarts

**Affiliations:** 1 Department of Bariatric Surgery, Vitalys Part of Rijnstate Hospital, 6815 AD Arnhem, The Netherlands; 2 Divison of Human Nutrition and Health, Wageningen University, 6708 WE Wageningen, The Netherlands

**Keywords:** Bariatric Surgery, Metabolic Surgery, Sleeve Gastrectomy, SG, Deficiencies, Micronutrients, Vitamins, Minerals, Supplementation

## Abstract

**Purpose:**

Lifelong daily multivitamin supplementation is highly recommended after sleeve gastrectomy (SG). Based on previous research, a specialized multivitamin supplement (MVS) for SG patients was developed and optimized (WLS Optimum 1.0 and 2.0). This study presents its mid-term effectives and compares micronutrient status of SG patients using this specialized MVS to users of standard MVS (sMVS) and non-users of multivitamin supplementation during the first three years post-surgery.

**Materials and Methods:**

Of the 226 participants that were included at baseline, yearly follow-up blood tests were completed by 193 participants (85%) at 12 months, 176 participants (78%) at 24 months, and 140 participants (62%) at 36 months of follow-up. At each time point, participants were divided into four groups: (1) Optimum 1.0, (2) Optimum 2.0, (3) sMVS, and (4) non-users. Serum concentrations (linear mixed-effects models) and the prevalence of micronutrient deficiencies (chi-square tests) during follow-up were compared between the groups.

**Results:**

Users of specialized MVS (Optimum 1.0 and 2.0) had higher serum concentrations of hemoglobin, folic acid, and vitamin D compared to sMVS users and non-users during follow-up. Serum concentrations of vitamin B12 and (corrected) calcium were also higher in specialized MVS users than in non-users. Overall, fewer deficiencies for folic acid and vitamin D were observed in the Optimum groups.

**Conclusion:**

Although the perfect multivitamin supplement for all SG patients does not exist, WLS Optimum was more effective in sustaining normal serum concentrations than standard, over-the-counter supplementation. Non-users of MVS presented with most micronutrient deficiencies and will evidently develop poor nutritional status on the longer term.

**Graphical abstract:**

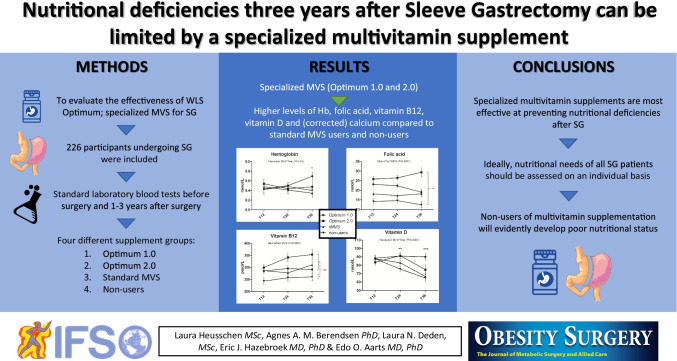

**Supplementary Information:**

The online version contains supplementary material available at 10.1007/s11695-022-06256-w.

## Introduction

During the past decade, the laparoscopic sleeve gastrectomy (SG) has become the most performed metabolic procedure worldwide, accounting for about 50% of all registered procedures [1]. While SG is primarily considered a restrictive procedure, the reduction in gastric acid production and intrinsic factor secretion due to removal of a large part of the stomach may also affect absorption of micronutrients [2]. Contrary to initial belief, similar rates of long-term nutritional deficiencies are found in SG patients when compared to patients that have undergone Roux-en-Y gastric bypass, even though the intestinal surface area remains intact following SG [3–6]. Micronutrient deficiencies for vitamin D, vitamin B12, and iron as well as elevated parathyroid hormone (PTH) levels have been reported up to 5 years after SG [7–10]. For that reason, a specialized multivitamin supplement specifically targeted to the needs of SG patients was developed (WLS Optimum; FitForMe, Rotterdam, the Netherlands). The composition of WLS Optimum was previously evaluated in a randomized controlled trial and optimized afterwards [11, 12]. The first version of WLS Optimum (1.0) was effective in reducing the prevalence of anemia and improving serum levels of folic acid, PTH, and vitamin B1 1 year after SG in comparison to a standard, over-the-counter multivitamin supplement (sMVS) [11]. The optimized version of WLS Optimum (2.0) additionally improved serum levels of vitamin B12, vitamin B6, and zinc, and resulted in less deficiencies for vitamin B12 and phosphate during the first year after SG, in comparison to WLS Optimum 1.0 [12]. However, the effectiveness of such specialized MVS on the longer term after SG is still unknown. In addition to this, compliance to supplementation regimes appears to be poor after bariatric surgery and a part of the patients discontinue the use of (specialized) MVS several years after surgery [13–16]. Research reporting on nutritional status of non-users of MVS following SG is limited. Therefore, the aim of this study was to evaluate micronutrient status of SG patients using specialized MVS (WLS Optimum 1.0, WLS Optimum 2.0) compared to sMVS and non-users during the first three years after surgery.

## Methods

### Study Design and Participants

The present study uses follow-up data of two former studies investigating the specialized multivitamin supplement WLS Optimum; the VITAAL I and VITAAL II study [11, 12].

VITAAL I was a randomized controlled trial aimed to evaluate the effectiveness of the first version of WLS Optimum (Optimum 1.0) [11]. Included patients received Optimum 1.0 (intervention group) or a standard, over-the-counter multivitamin supplement (sMVS; control group) for 12 months. After the intervention period, the blinded component of the study was terminated. During follow-up, standard extensive blood tests were performed yearly up to 3 years post-SG. VITAAL II was designed to evaluate the effectiveness of the improved version of the WLS Optimum supplement (Optimum 2.0). In contrast to the initial RCT, there was no control group in this study [12]. All participants received Optimum 2.0 and were instructed to use this supplement on a daily basis for 12 months. Similarly, all patients were invited to complete their yearly follow-up blood tests up to 3 years post-SG.

Both study protocols were approved by the Medical Ethics Review Committee of the Radboud University Medical Centre and the Local Ethical Committee of Rijnstate Hospital Arnhem, and were conducted in concordance with the principles of the Declaration of Helsinki. The VITAAL I study was registered at the clinical trials registry of the National Institutes of Health (ClinicalTrials.gov; identifier NCT01609387).

**Fig. 1:**
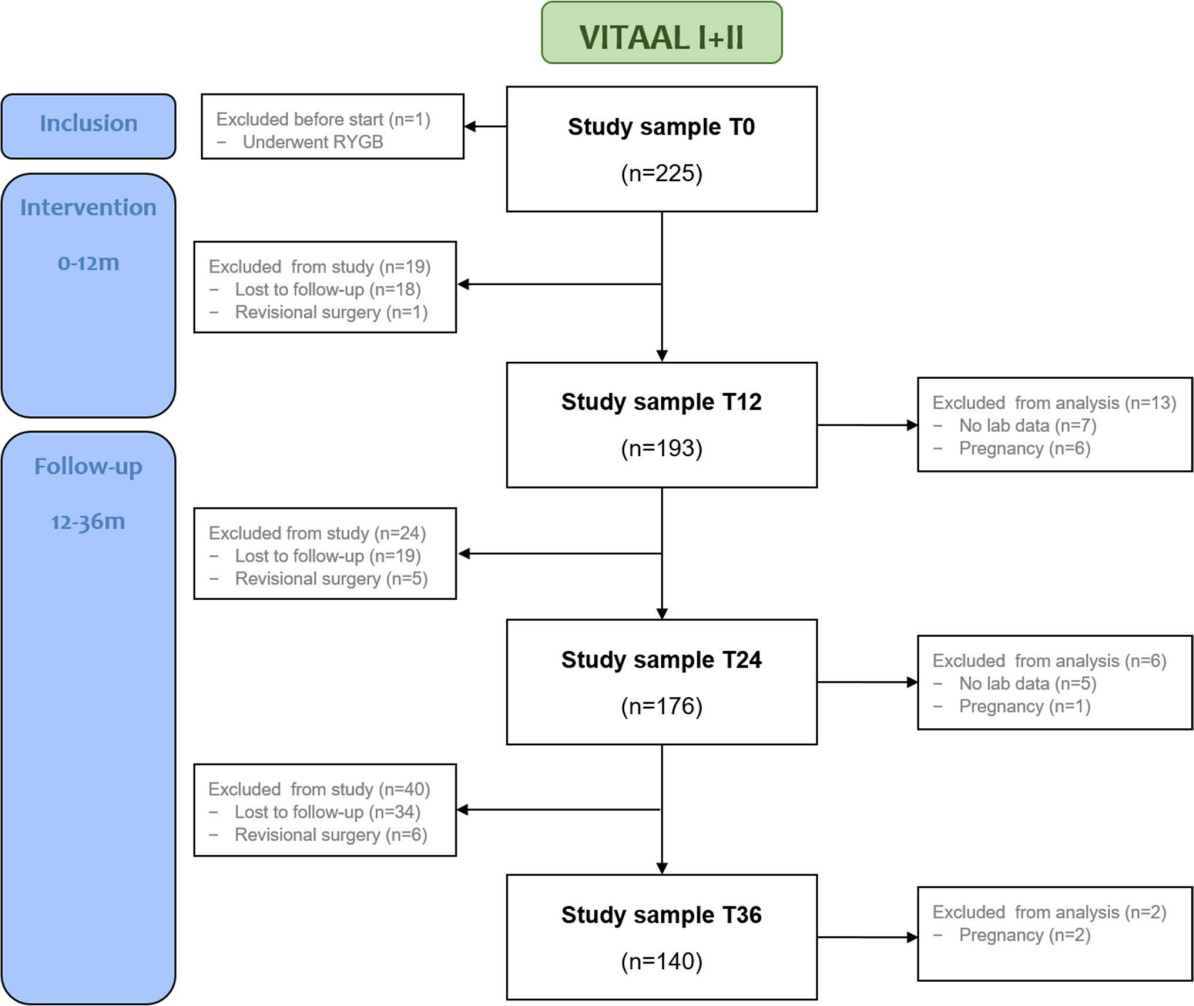
Flowchart of the study sample for data analysis at baseline (T0), after the 12-month intervention period (T12), and at 24 and 36 months of follow-up (T24, T36)

A total of 226 participants were included in the VITAAL I (*n* = 150) and VITAAL II study (*n* = 76). During the follow-up period (12–36 months), 53 participants were lost to follow-up, and 11 underwent revisional surgery. Additionally, participants with missing laboratory data (*n* = 12) or known pregnancy (*n* = 9) at the time of follow-up were excluded from the analyses (Fig. 1). A detailed flowchart of the individual studies can be found in Supplementary Fig. 1. For the present study, the final study sample for data analysis consisted of 193 participants (85%) at 12 months, 176 participants (78%) at 24 months, and 140 participants (62%) at 36 months of follow-up.

### Data Collection

#### Demographic Information

Socio-demographic (age, sex) and health-related information (anthropometrics, comorbidities) were collected during standard follow-up visits at the hospital. Bodyweight was measured to the nearest 0.1 kg with a digital weighing scale (Tanita BC-420MA, Tokyo, Japan), after removal of heavy clothing and shoes. Height was measured in standing position with a wall-mounted stadiometer (Seca 206, Hamburg, Germany). BMI was calculated as weight (kg) divided by squared height (m^2^). Total body weight loss (TWL) was calculated as weight loss divided by weight before surgery, multiplied by 100%. Excess weight loss (EWL) was calculated as weight loss divided by excess weight before surgery (based on ideal body weight at BMI 25 kg/m^2^), multiplied by 100%.

#### Supplementation Use

Self-reported information on the use of multivitamin supplementation (brand, content, and compliance) at each follow-up visit were obtained via medical chart review and participants were divided into four different treatment modalities: (1) Optimum 1.0 (2), Optimum 2.0 (3), sMVS, and (4) non-users.

The composition of WLS Optimum 1.0 and Optimum 2.0 is shown in Table 1. Compared to the first version, Optimum 2.0 contained higher doses of elementary iron, folic acid, vitamin B12, vitamin B1, copper and zinc, and a lower dose of vitamin A. Moreover, it is important to note that after the 12-month study period of the VITAAL II study, the dose of vitamin D in Optimum 2.0 was increased from 7.5 μg (150% RDA) to 75 μg (1500% RDA). During follow-up, all participants received the supplement with this high dose of vitamin D. Both supplements were dosed as one capsule per day.

**Table 1:** Content of WLS Optimum 1.0 and Optimum 2.0

Micronutrients	Optimum 1.0		Optimum 2.0	
	Dose	RDA (%)	Dose	RDA (%)
*Vitamins*				
Vitamin A, mg	1.00	125.0	0.80	100.0
Vitamin B1, mg	2.00	182.0	2.75	250.0
Vitamin B2, mg	2.00	143.0	2.00	143.0
Vitamin B3, mg	25.00	156.0	25.00	156.0
Vitamin B5, mg	9.00	150.0	9.00	150.0
Vitamin B6, mg	2.00	143.0	2.00	143.0
Biotin, μg	150.00	300.0	150.00	300.0
Folic acid, μg	300.00	150.0	500.00	250.0
Vitamin B12, μg	10.00	400.0	100.00	4000.0
Vitamin C, mg	100.00	125.0	100.00	125.0
Vitamin D, μg	7.50	150.0	75.00^1^	1500.0
Vitamin E, mg	12.00	100.0	12.00	100.0
Vitamin K1, μg	90.00	120.0	-	-
*Minerals*				
Chrome, μg	40.00	100.0	40.00	100.0
Iron, mg	21.00	150.0	28.00	200.0
Iodine, μg	150.00	100.0	150.00	100.0
Copper, mg	1.00	100.0	1.90	190.0
Magnesium, mg	30.00	8.0	-	-
Manganese, mg	3.00	150.0	3.00	150.0
Molybdenum, μg	50.00	100.0	50.00	100.0
Selenium, μg	55.00	100.0	55.00	100.0
Zinc, mg	15.00	150.0	28.00	280.0

*RDA* recommended daily allowance

^1^After the 12-month study period, the dose of vitamin D in WLS Optimum 2.0 was increased from 7.5 μg (150% RDA) to 75 μg (1500% RDA)

sMVS were defined as standard, over-the-counter supplements that usually contain nutrients in amounts of 100% of the RDA. In addition, participants were advised to take calcium/vitamin D3 500 mg/800 IE supplements two times a day as part of the standard treatment post-SG.

Furthermore, data on the use of additional supplementation (e.g., vitamin B12 injections) were also retrieved from the medical records. When additional supplementation was used, data of subsequent serum concentrations for that micronutrient were removed from the analyses to prevent biased estimates.

#### Laboratory Blood Tests

Standard laboratory blood tests were performed at baseline (T0, pre-surgery), after the 12-month intervention period (T12) and at 24 months (T24) and 36 months (T36) of follow-up. Blood serum and plasma were collected by venipuncture at all timepoints. The following blood parameters were measured on random access analyzers: hemoglobin, mean corpuscular volume (MCV; XN-10 Sysmex); ferritin, folic acid, vitamin B12, 25-OH vitamin D, PTH (Modular E170, Roche); and calcium, albumin (Modular P800, Roche). Calcium levels were corrected for albumin using the following equation: Ca_corr_ = total calcium − (0.025 × albumin) + 1. A deficiency was defined as a serum level below the local reference value at the time of blood collection (Table 2).

**Table 2:** Reference ranges of the evaluated micronutrients

Micronutrients	Reference range
Hemoglobin	Male: 8.4–10.8 mmol/L Female: 7.4–9.9 mmol/L
MCV	80–100 fL
Ferritin	20–300 ng/mL
Folic acid^1^	6–28 nmol/L
Vitamin B12	200–570 pmol/L
Vitamin D	> 50 nmol/L
PTH^2^	1.3–6.8 pmol/L
Calcium^3^	2.10–2.55 mmol/L
Albumin	35–50 g/L

*MCV* mean corpuscular volume, *PTH* parathyroid hormone

^1^Reference range for the assay in the VITAAL II study was 5–35 nmol/L at 24 months and > 12.2 nmol/L at 36 months

^2^Reference range for the assay in the VITAAL II study was 1.96–9.33 pmol/L at 36 months

^3^Reference range for the assay in the VITAAL II study was 2.20–2.55 mmol/L at 24 months and 2.08–2.65 mmol/L at 36 months

### Statistical Analyses

General characteristics of the study population are reported as median and interquartile range [Q1–Q3] for continuous data and as frequency (percentage) for categorical data. Differences in preoperative characteristics between the study population at baseline and during follow-up were analyzed using the Kruskal–Wallis test for continuous data and chi-square tests for categorical data (or Fisher’s exact test when > 20% of expected counts were < 5). Serum concentrations during follow-up were analyzed using a mixed-effects model accounting for the fixed effects of MVS (Optimum 1.0; Optimum 2.0; sMVS; non-users) and time (T12; T24; T36), and their interaction term, plus the random effect of the participants. Time entered the model as a repeated measure using a first-order autoregressive structure with heterogeneous variances. BMI was used as a covariate, entering the model as a fixed effect. Results are presented as estimated marginal mean ± standard error. Means and standard deviations of the original serum data at the different time points can be found in Supplementary table 1. The prevalence of deficiencies at each time point was analyzed using chi-square tests (or Fisher’s exact test when > 20% of expected counts were < 5). In case of a significant main effect, post hoc pairwise comparisons were performed. *P*-values of post hoc tests were adjusted using the Bonferroni correction.

All statistical analyses were performed using IBM SPSS Statistics 25 for Windows (IBM Corp., Armonk USA). A two-sided *P*-value below 0.05 was considered statistically significant.

## Results

Preoperative characteristics of the study population at baseline (*n* = 225) were comparable to those of the study population at T12 (*n* = 193), T24 (*n* = 176), and T36 (*n* = 140) with respect to sex, age, BMI, and comorbidities (Table 3). At baseline, 76% of the participants was female, with a median age of 38.4 [29.0–47.5] years and a median BMI of 45.5 [40.6–54.1] kg/m^2^. During follow-up, median BMI declined to 30.8 [26.7–36.6] kg/m^2^ at T24 and 30.3 [27.2–35.8] kg/m^2^ at T36, with a median TWL of 32.1 [24.1–38.8]% and 30.0 [22.1–35.5]%, respectively.

**Table 3:** General characteristics of the study sample at baseline (T0) and at 12, 24 and 36 months of follow-up (T12, T24, T36)

	T0 (*n* = 225)	T12 (*n* = 193)	T24 (*n* = 176)	T36 (*n* = 140)
Sex (female)	171 (76.0)	145 (75.1)	136 (77.3)	105 (75.0)
Age before surgery (years)	38.4 [29.0–47.5]	39.2 [29.5–47.8]	39.3 [30.0–47.9]	40.9 [30.1–49.0]
Body weight before surgery (kg)	135.7 [119.9–162.4]	135.7 [120.7–164.7]	135.2 [119.8–162.5]	131.0 [117.0–154.8]
BMI before surgery (kg/m^2^)	45.5 [40.6–54.1]	44.2 [40.6–54.9]	44.9 [40.6–54.5]	42.4 [40.1–52.8]
Previous adjustable gastric band	12 (5.3)	11 (5.7)	9 (5.1)	8 (5.7)
Comorbidities before surgery				
Diabetes mellitus type 2	25 (11.1)	23 (11.9)	21 (11.9)	19 (13.6)
Hypertension	57 (25.3)	50 (25.9)	46 (26.1)	39 (27.9)
Dyslipidemia	21 (9.3)	19 (9.8)	17 (9.7)	18 (12.9)
OSAS	22 (9.8)	21 (10.9)	22 (12.5)	18 (12.9)
BMI after surgery (kg/m^2^)^1^	-	30.5 [26.9–37.5]	30.8 [26.7–36.6]	30.3 [27.2–35.8]
EWL after surgery (%)^1^	-	69.2 [54.1–88.0]	70.5 [55.4–91.7]	67.2 [53.5–87.7]
TWL after surgery (%)^1^	-	30.9 [25.6–37.6]	32.1 [24.1–38.8]	30.0 [22.1–35.5]

Data are presented as median [Q1–Q3] and frequencies (percentages)

*BMI* body mass index, *OSAS* obstructive sleep apnea syndrome, *EWL* excess weight loss, *TWL* total weight loss

^1^Missing for *n* = 2 at T12, *n* = 2 at T24, and *n* = 5 at T36

After the intervention period (T12), 23% of the study population used Optimum 1.0, 18% used Optimum 2.0, 46% used a sMVS, and 12% of the participants were non-users. During follow-up, Optimum 1.0 was used by 37% at T24 and 33% at T36, Optimum 2.0 by 18% and 16%, and sMVS by 27% and 28%. The group of non-users increased from 18% at T24 to 24% at T36.

### Micronutrient Serum Concentrations

**Fig. 2:**
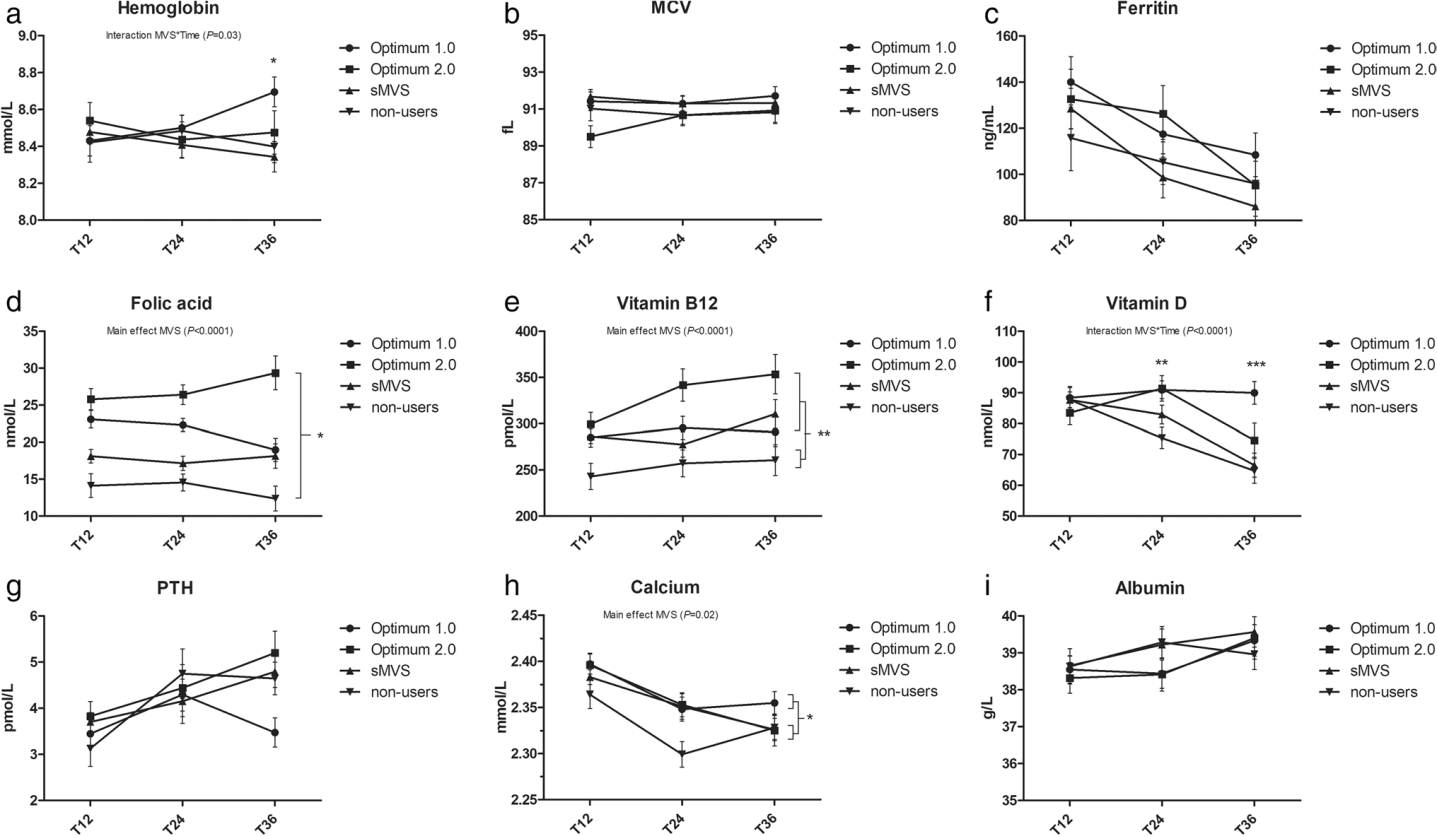
Serum concentrations for all groups after the 12-month intervention period (T12), and at 24 and 36 months of follow-up (T24, T36). Lines depict estimated marginal means ± standard errors (error bars). **a** Hemoglobin. *Significantly higher serum levels for Optimum 1.0 compared to sMVS and non-users at T36 (*P* < 0.05). **b** MCV. **c** Ferritin. **d** Folic acid. *Significantly different serum levels between all groups (*P* < 0.05). **e** Vitamin B12. **Significantly lower serum levels for non-users compared to all other groups (*P* < 0.01). **f** Vitamin D. **Significantly higher serum levels for Optimum 1.0 and Optimum 2.0 compared to non-users at T24 (*P* < 0.01). ***Significantly higher serum levels for Optimum 1.0 compared to non-users and sMVS at T36 (*P* < 0.001). **g** PTH. **h** (Corrected) calcium. *Significantly higher serum levels for Optimum 1.0 compared to non-users (*P* = 0.02). **i** Albumin

Changes in serum concentrations over time for the four groups are shown in Fig. 2.

Significant main effects of MVS were found for folic acid, vitamin B12, and corrected calcium. Serum folic acid concentrations were highest in Optimum 2.0 users (26.2 ± 1.2 nmol/L) followed by Optimum 1.0 users (21.9 ± 0.9 nmol/L) and sMVS users (17.6 ± 0.8 nmol/L), and lowest in the non-users (13.8 ± 1.0 nmol/L), *P* < 0.05 for all. Serum vitamin B12 concentrations were also lowest in non-users (253.9 ± 11.3 pmol/L) compared to all other groups (*P* < 0.01 for all). Corrected calcium concentrations were higher in Optimum 1.0 users (2.37 ± 0.01 mmol/L) than in non-users (2.33 ± 0.01 mmol/L), *P* = 0.02.

For hemoglobin and vitamin D, there was a significant interaction between MVS and time, indicating that serum hemoglobin and vitamin D concentrations differed significantly over time between the four groups. Serum hemoglobin concentrations were comparable between all groups at T12 and T24, but higher in Optimum 1.0 users (8.7 ± 0.08 mmol/L) at T36 compared to sMVS users (8.3 ± 0.08 mmol/L, *P* < 0.01) and non-users (8.4 ± 0.09 mmol/L, *P* = 0.04). For vitamin D, serum concentrations were similar for all groups at T12 and higher in Optimum 1.0 users (90.9 ± 2.9 nmol/L) and Optimum 2.0 users (91.4 ± 4.2 nmol/L) than in non-users (75.4 ± 3.5 nmol/L, *P* < 0.01 for both) at T24. At T36, serum vitamin D concentrations were also higher in the Optimum 1.0 group (90.0 ± 3.7 nmol/L) compared to the group of non-users (64.7 ± 4.1 nmol/L, *P* < 0.001) as well as the sMVS group (66.5 ± 3.9 nmol/L, *P* < 0.001). Serum vitamin D concentrations of the Optimum 2.0 group (74.6 ± 5.5 nmol/L) were no longer different from the other groups at T36.

No differences between the groups were observed for MCV, ferritin, PTH, and albumin.

### Micronutrient Deficiencies

During follow-up, the number of deficiencies for folic acid (T24, T36) and vitamin D (T36) were significantly different between the four groups (*P* < 0.01 for all, Table 4). For folic acid, the number of deficiencies was lower in the Optimum 1.0 group compared to the group of non-users at both T24 (1.6% vs 21.9%, *P* = 0.01) and T36 (0% vs 24.2%, *P* < 0.01). The number of vitamin D deficiencies at T36 was also lowest in the Optimum 1.0 group (2.2%), compared to all other groups (respectively 26.3%, 35.1% and 32.3% for Optimum 2.0, sMVS and non-users, *P* < 0.05 for all). At T36, the prevalence of vitamin B12 deficiency tended to be lower in the Optimum 2.0 group with no deficiencies observed in this group, compared to 12.1% in the Optimum 1.0 group, 9.4% in the sMVS group, and 20.8% in the group of non-users (*P* > 0.05).

**Table 4:** Prevalence of deficiencies at baseline (T0), after the 12-month intervention period (T12), and at 24 and 36 months of follow-up (T24, T36) for the four groups

Serum variables	Type of MVS	*n*	T0	*n*	T12	*n*	T24	*n*	T36
Hemoglobin	Optimum 1.0	69	4 (5.8)	45	2 (4.4)	65	6 (9.2)	46	3 (6.5)
	Optimum 2.0	75	3 (4.0)	35	2 (5.7)	31	4 (12.9)	22	4 (18.2)
	sMVS	69	4 (5.8)	88	13 (14.8)	48	3 (6.3)	39	4 (10.3)
	Non-users	*-*		24	2 (8.3)	31	1 (3.2)	32	1 (3.1)
MCV	Optimum 1.0	68	1 (1.5)	45	0 (0.0)	65	2 (3.1)	46	0 (0.0)
	Optimum 2.0	75	1 (1.3)	35	0 (0.0)	31	0 (0.0)	22	1 (4.5)
	sMVS	68	2 (2.9)	88	2 (2.3)	48	0 (0.0)	39	0 (0.0)
	Non-users	*-*		24	0 (0.0)	31	0 (0.0)	32	0 (0.0)
Ferritin	Optimum 1.0	69	2 (2.9)	44	1 (2.3)	61	6 (9.8)	42	5 (11.9)
	Optimum 2.0	75	1 (1.3)	34	1 (2.9)	28	2 (7.1)	20	2 (10.0)
	sMVS	70	3 (4.3)	88	5 (5.7)	47	6 (12.8)	38	5 (13.2)
	Non-users	*-*		24	4 (16.7)	32	5 (15.6)	31	4 (12.9)
Folic acid	Optimum 1.0	68	2 (2.9)	45	1 (2.2)	63	1 (1.6)^a^	43	0 (0)^a^
	Optimum 2.0	75	0 (0.0)	34	1 (2.9)	29	1 (3.4)^a,b^	19	1 (5.3)^a,b^
	sMVS	69	4 (5.8)	89	6 (6.7)	48	5 (10.4)^a,b^	37	5 (13.5)^a,b^
	Non-users	*-*		24	4 (16.7)	32	7 (21.9)^b^	33	8 (24.2)^b^
Vitamin B12	Optimum 1.0	67	1 (1.5)	40	8 (20.0)^a,b^	51	9 (17.6)	33	4 (12.1)
	Optimum 2.0	71	1 (1.4)	29	1 (3.4)^a^	25	3 (12.0)	19	0 (0.0)
	sMVS	70	0 (0.0)	86	16 (18.6)^a,b^	34	6 (17.6)	32	3 (9.4)
	Non-users	-		22	10 (45.5)^b^	28	9 (32.1)	24	5 (20.8)
Vitamin D	Optimum 1.0	69	51 (73.9)^a^	44	3 (6.8)	63	4 (6.3)	45	1 (2.2)^a^
	Optimum 2.0	7*5*	29 (38.7)^b^	35	1 (2.9)	31	1 (3.2)	19	5 (26.3)^b^
	sMVS	70	54 (77.1)^a^	87	9 (10.3)	47	6 (12.8)	37	13 (35.1)^b^
	Non-users	*-*		24	1 (4.2)	32	7 (21.9)	31	10 (32.3)^b^
PTH^1^	Optimum 1.0	69	7 (10.1)^a,b^	44	3 (6.8)	33	2 (6.1)	44	1 (2.3)
	Optimum 2.0	75	2 (2.7)^b^	34	3 (8.8)	30	2 (6.7)	19	0 (0.0)
	sMVS	70	10 (14.3)^a^	88	7 (8.0)	33	2 (6.1)	37	2 (5.4)
	Non-users	-		23	1 (4.3)	25	3 (12.0)	33	3 (9.1)
Calcium^2^	Optimum 1.0	62	1 (1.6)^a,b^	44	0 (0.0)	31	0 (0.0)	34	1 (2.9)
	Optimum 2.0	66	6 (9.1)^b^	34	1 (2.9)	31	0 (0.0)	20	0 (0.0)
	sMVS	68	0 (0.0)^a^	88	0 (0.0)	28	1 (3.6)	35	2 (5.7)
	Non-users	*-*		23	0 (0.0)	26	3 (11.5)	32	1 (3.1)
Albumin	Optimum 1.0	63	9 (14.3)	44	4 (9.1)	31	5 (16.1)	34	2 (5.9)
	Optimum 2.0	66	5 (7.6)	34	4 (11.8)	31	0 (0.0)	20	0 (0.0)
	sMVS	68	7 (10.3)	89	9 (10.1)	28	2 (7.1)	35	0 (0.0)
	Non-users	*-*		23	0 (0.0)	26	2 (7.7)	32	2 (9.4)

Data are presented as frequencies (percentages)

*MVS* multivitamin supplement, *sMVS* standard multivitamin supplement, *MCV* mean corpuscular volume, *PTH* parathyroid hormone

^1^Elevated PTH levels

^2^Corrected for albumin levels (total calcium − (0.025 × albumin) + 1)

Different letters denote significant differences between groups (*P* < 0.05)

Overall, the number of participants with one or more micronutrient deficiencies during follow-up was markedly lower in the Optimum 1.0 (32.4%) and Optimum 2.0 group (28.3%), than in the sMVS group (49.4%) and the group of non-users (66.2%), *P* < 0.001. For the Optimum users, anemia and deficiencies for vitamin B12 (Optimum 1.0) and vitamin D (Optimum 2.0) were most prevalent whereas, in the group of sMVS and non-users, deficiencies for folic acid, vitamin B12, and vitamin D were most common.

### Elevated Serum Levels

Elevated serum levels during follow-up were more prevalent in Optimum users than in sMVS and non-users. At T24, serum ferritin levels above the normal range (> 300 ng/mL) were observed in 6.6% and 21.4% of the Optimum 1.0 and 2.0 users, versus 0% and 3.1% of the sMVS-users and non-users (*P* < 0.01). At T36, the prevalence of elevated serum ferritin levels was no longer significantly different between the groups as it decreased to 5.0% in the Optimum 2.0 group. Serum vitamin B12 levels above the normal range (> 600 pmol/L) were mostly observed in the Optimum 2.0 group at both timepoints (12.0%, 15.8%), followed by the Optimum 1.0 users (2.0%, 0%) and sMVS users (0%, 3.1%) (*P* < 0.05 for both). There were no elevated serum levels for vitamin B12 observed in the non-users group.

## Discussion

Despite previous research and multiple guidelines, no multivitamin supplement has been able to consistently sustain normal serum concentrations for micronutrients. The present study found that users of specialized MVS (WLS Optimum 1.0 and 2.0) had higher serum concentrations of hemoglobin, folic acid, vitamin B12, vitamin D, and corrected calcium compared to sMVS users and non-users 3 years after SG. Similar trends were found for ferritin, although not statistically significant. Overall, least micronutrient deficiencies were also found for users of specialized MVS, followed by sMVS users. Non-users presented with the most deficiencies as well as the lowest serum concentrations for almost all micronutrients.

Over time, supplement use varied and adherence to MVS declined with less than half of the participants consistently using the same MVS throughout follow-up and the percentage of non-users increasing up to 24% at 3 years after SG. This is in line with other research on adherence to supplementation regimes after bariatric surgery with (self-reported) compliance rates ranging between 37 and 93% up to 5 years post-surgery [7, 14, 15, 17–20]. Besides commonly reported barriers as gastro-intestinal side effects, bad taste/smell/size, and high cost [13, 15, 19], some patients believe that their diet provides sufficient micronutrients and therefore do not feel the need to use MVS [15, 19]. This is concerning as we found that about 66% of the non-users in this study presented with one or more nutrient deficiencies during follow-up, whereas this was only about 30% in the groups that used a specialized MVS. Moreover, serum concentrations of almost all evaluated micronutrients were lowest in the group of non-users throughout follow-up. This in line with a study of Dagan et al. including 77 SG patients that showed that adherence to multivitamin supplementation at 12 months was significantly associated with higher serum levels of hemoglobin, iron, folic acid, and vitamins B12 and D [14]. As with the general adherence to medical follow-up visits after bariatric surgery, compliance with post-surgery supplementation protocols tends to decrease with time from surgery [7, 13–16]. As a result, nutritional status may worsen over time. This reinforces the need for long-term nutritional follow-up and counseling while taking patients’ barriers related to supplementation use into account.

The increase in the level of folic acid (300 to 500 µg) and vitamin B12 (10 to 100 µg) between the first and second version of WLS Optimum was clearly reflected in higher serum levels for these micronutrients in the Optimum 2.0 group. For vitamin B12, this also resulted in fewer vitamin B12 deficiencies in the Optimum 2.0 group compared to the Optimum 1.0 group, although these findings did not reach statistical significance. In contrast, the tenfold increase in vitamin D (7.5 to 75 μg) did not consistently result in higher serum vitamin D concentrations in the Optimum 2.0 group throughout follow-up. At T24, Optimum 2.0 users showed higher serum vitamin D concentrations and fewer vitamin D deficiencies than Optimum 1.0 users, while the opposite was observed at T36 with 26% of the Optimum 2.0 users being vitamin D deficient compared to 2% of the Optimum 1.0 users. This could have been caused by seasonal differences in the timing of follow-up measurements as vitamin D levels are highly influenced by the amount of sun exposure [21]. The number of patients completing their follow-up measurements between November and April, which is the period of low sun exposure in the northern latitudes [22], was indeed markedly higher in the Optimum 2.0 group compared to the Optimum 1.0 group, especially at 3 years of follow-up (77% vs 50%). Furthermore, a difference in compliance to the standard postoperative calcium/vitamin D3 supplementation regimen could have also impacted our findings with regard to vitamin D status.

The level of elementary iron in WLS Optimum was increased from 21 mg in Optimum 1.0 to 28 mg in Optimum 2.0, but this did not result in fewer ferritin deficiencies in the latter group. In fact, the number of ferritin deficiencies was comparable between all groups, ranging from 7 to 16% at 2 years and from 10 to 13% at 3 years of follow-up, which is lower than reported in previous literature (17–59% at 2–4 years after SG) [8, 9, 20, 23–25]. Although serum ferritin concentrations were highest in Optimum 2.0 users at each specific time point, we still observed a decrease in serum levels over time in this group. In a recent systematic review and meta-analysis including 82 studies on longitudinal changes in micronutrient status after bariatric surgery, ferritin levels also decreased at 24 months after SG despite supplementation per guidelines [26]. The observed decrease in serum ferritin concentration might have been secondary to depletion of the body’s iron reserves after bariatric surgery as the prevalence of anemia in the Optimum 2.0 group increased from 13% at T24 to 18% at T36, suggesting that the body’s iron stores were not sufficient to prevent patients from developing iron-deficiency anemia. This could indicate that 28 mg elementary iron is not sufficient to keep serum ferritin concentrations stable on the longer term, particularly in patients who are at higher risk such as premenopausal women. Alternate day dosing of iron could be an alternative solution as it significantly increases iron absorption and results in a lower incidence of gastro-intestinal side effects compared with dosing iron every day [27, 28]. On the other hand, elevated serum ferritin levels were most frequently observed in the Optimum 2.0 group, showing the complexity of micronutrient supplementation.

Overall, elevated serum levels during follow-up were more prevalent in Optimum users than in sMVS users and non-users. Yet, it is important to note that certain nutrients such as folic acid are highly sensitive to recent intake [29, 30]. Healthcare practitioners may therefore suggest fasting from MVS intake up to 12–24 h prior to a blood test. Regarding folic acid, the upper assay limit of 45 nmol/L also hindered to assess whether plasma levels were extremely elevated. However, this was the case in only 15 patients. Clinical manifestations of toxicity have not been actively investigated in the present study, but no adverse events due to hypervitaminosis were reported. Yet, toxicity on the long term is largely unknown. For example, high plasma concentrations of vitamin B12 have been associated with increased risks of certain types of cancer [31, 32] and all-cause mortality [33]. Observational data that evaluate the long-term consequences of supplementing such high doses in this patient population are needed.

The main strength of this study is that it is one of the first that evaluates mid-term micronutrient status after SG, while discriminating between different types of MVS. By using mixed-effects models analysis, we approximated the longitudinal effect of MVS use as much as possible but we could not prevent potential cross-over effects resulting from switching between different MVS formulations in-between time points. As only a small number of participants consistently used the same MVS throughout the follow-up period, we were not able to determine the efficacy of supplementation within these subgroups and to take compliance into account. Other limitations include the changes in composition of WLS Optimum over time and the high number of participants who were lost to follow-up which resulted in a lack of statistical power, particularly for the analyses on nutrient deficiencies.

## Conclusion

Evidently, there is no one-size-fits-all when it comes to multivitamin supplementation after sleeve gastrectomy. Even specialized supplementation that is specifically targeted to the needs of this patient population could not completely prevent micronutrient deficiencies from occurring. Nevertheless, daily use of specialized MVS is markedly more effective in sustaining normal serum concentrations than standard, over-the-counter supplementation. Non-users of MVS presented with most micronutrient deficiencies and will evidently develop poor nutritional status on the longer term, reinforcing the need of long-term nutritional follow-up and counseling while taking patients’ barriers related to supplementation use into account.

## Supplementary Information

Below is the link to the electronic supplementary material.

Supplementary file1 (DOCX 266 KB)
