# Supplementary material for: Nutritional Deficiencies 3 Years After Sleeve Gastrectomy Can Be Limited by a Specialized Multivitamin Supplement
Source: Obes Surg. 2022 Aug 26;32(11):3561–70. doi: 10.1007/s11695-022-06256-w (PMC9613574; doi:10.1007/s11695-022-06256-w)
Supplement: Supplementary file 1 — Supplementary file1 (DOCX 266 KB) [file 11695_2022_6256_MOESM1_ESM.docx]

**Supplementary Table 1.** Serum concentrations at baseline (T0), after the 12-month intervention period (T12), and at 24 and 36 months of follow-up (T24, T36) for the four groups.

| Serum variables | Type of MVS | *n* | **T0** | | *n* | **T12** | | *n* | **T24** | | *n* | **T36** | |
| --- | --- | --- | --- | --- | --- | --- | --- | --- | --- | --- | --- | --- | --- |
| **Hemoglobin**  (mmol/L) | Optimum 1.0 | *69* | 8.7 | ±0.8 | *45* | 8.6 | ±0.7 | *65* | 8.5 | ±0.7 | *46* | 8.6 | ±0.7 |
|  | Optimum 2.0 | *75* | 8.8 | ±0.8 | *35* | 8.7 | ±0.8 | *31* | 8.4 | ±0.7 | *22* | 8.6 | ±0.9 |
|  | sMVS | *69* | 8.8 | ±0.7 | *88* | 8.4 | ±0.7 | *48* | 8.3 | ±0.7 | *39* | 8.4 | ±0.7 |
|  | Non-users | *-* |  |  | *24* | 8.4 | ±0.7 | *31* | 8.6 | ±0.5 | *32* | 8.5 | ±0.6 |
| **MCV**  (fL) | Optimum 1.0 | *68* | 88.7 | ±4.1 | *45* | 91.6 | ±3.8 | *65* | 91.5 | ±4.5 | *46* | 91.6 | ±4.9 |
|  | Optimum 2.0 | *75* | 88.8 | ±3.8 | *35* | 89.6 | ±3.7 | *31* | 90.8 | ±3.2 | *22* | 90.1 | ±4.1 |
|  | sMVS | *68* | 88.9 | ±4.7 | *88* | 91.6 | ±4.7 | *48* | 91.5 | ±3.8 | *39* | 91.4 | ±4.4 |
|  | Non-users | *-* |  |  | *24* | 91.5 | ±4.7 | *31* | 90.1 | ±3.7 | *32* | 90.9 | ±3.8 |
| **Ferritin**  (ng/mL) | Optimum 1.0 | *69* | 127.6 | ±96.4 | *44* | 144.9 | ±111.6 | *61* | 123.6 | ±96.8 | *42* | 100.8 | ±86.5 |
|  | Optimum 2.0 | *75* | 119.8 | ±99.3 | *34* | 151.0 | ±112.6 | *28* | 152.8 | ±134.0 | *20* | 127.6 | ±111.6 |
|  | sMVS | *70* | 128.8 | ±97.7 | *88* | 128.0 | ±78.2 | *47* | 91.5 | ±68.4 | *38* | 94.6 | ±88.1 |
|  | Non-users | *-* |  |  | *24* | 97.5 | ±77.3 | *32* | 99.6 | ±80.0 | *31* | 94.9 | ±93.2 |
| **Folic acid**  (nmol/L) | Optimum 1.0 | *68* | 16.6 | ±6.7 | *45* | 24.6 | ±9.9 | *63* | 23.5 | ±9.3 | *43* | 21.9 | ±9.1 |
|  | Optimum 2.0 | *75* | 14.9 | ±6.2 | *34* | 26.6 | ±14.1 | *29* | 28.3 | ±13.1 | *19* | 30.8 | ±14.4 |
|  | sMVS | *69* | 16.7 | ±6.0 | *89* | 17.9 | ±7.0 | *48* | 16.4 | ±7.9 | *37* | 18.3 | ±10.6 |
|  | Non-users | *-* |  |  | *24* | 11.8 | ±6.1 | *32* | 12.3 | ±4.8 | *33* | 10.9 | ±4.7 |
| **Vitamin B12**  (pmol/L) | Optimum 1.0 | *67* | 289.8 | ±96.4 | *40* | 279.4 | ±76.0 | *51* | 298.8 | ±101.3 | *33* | 310.8 | ±102.6 |
|  | Optimum 2.0 | *71* | 299.7 | ±95.6 | *29* | 322.2 | ±98.4 | *26* | 382.1 | ±176.7 | *19* | 403.3 | ±151.3 |
|  | sMVS | *70* | 315.9 | ±110.1 | *86* | 286.3 | ±83.9 | *34* | 297.0 | ±89.7 | *32* | 331.7 | ±120.2 |
|  | Non-users | - |  |  | *22* | 233.3 | ±84.4 | *28* | 232.1 | ±64.7 | *24* | 263.7 | ±72.0 |
| **Vitamin D**  (nmol/L) | Optimum 1.0 | *69* | 36.6 | ±21.8 | *44* | 91.0 | ±30.4 | *63* | 92.4 | ±27.6 | *45* | 92.2 | ±26.7 |
|  | Optimum 2.0 | *75* | 55.8 | ±24.7 | *35* | 88.5 | ±20.6 | *31* | 100.6 | ±31.8 | *19* | 84.7 | ±41.1 |
|  | sMVS | *70* | 34.0 | ±16.7 | *87* | 86.4 | ±29.6 | *47* | 84.0 | ±31.9 | *37* | 67.8 | ±32.9 |
|  | Non-users | *-* |  |  | *24* | 80.2 | ±27.8 | *32* | 69.6 | ±23.9 | *31* | 65.3 | ±29.1 |
| **PTH**  (pmol/L) | Optimum 1.0 | *69* | 3.7 | ±2.0 | *44* | 3.3 | ±2.1 | *33* | 4.5 | ±4.2 | *44* | 3.6 | ±1.6 |
|  | Optimum 2.0 | *75* | 3.3 | ±2.0 | *34* | 3.9 | ±2.2 | *30* | 4.5 | ±2.0 | *19* | 5.0 | ±1.2 |
|  | sMVS | *70* | 4.0 | ±2.5 | *88* | 3.8 | ±2.0 | *33* | 3.9 | ±1.7 | *37* | 4.7 | ±2.1 |
|  | Non-users | - |  |  | *23* | 3.2 | ±1.8 | *25* | 4.9 | ±2.5 | *33* | 4.6 | ±2.9 |
| **Calcium**^1^  mmol/L | Optimum 1.0 | *62* | 2.35 | ±0.11 | *44* | 2.40 | ±0.09 | *31* | 2.36 | ±0.09 | *34* | 2.36 | ±0.09 |
|  | Optimum 2.0 | *66* | 2.34 | ±0.10 | *34* | 2.39 | ±0.09 | *31* | 2.35 | ±0.07 | *20* | 2.32 | ±0.07 |
|  | sMVS | *68* | 2.35 | ±0.09 | *88* | 2.39 | ±0.08 | *28* | 2.35 | ±0.07 | *35* | 2.34 | ±0.09 |
|  | Non-users | *-* |  |  | *23* | 2.36 | ±0.06 | *26* | 2.29 | ±0.09 | *32* | 2.33 | ±0.09 |
| **Albumin**  (g/L) | Optimum 1.0 | *63* | 37.8 | ±3.8 | *44* | 38.7 | ±3.1 | *31* | 38.1 | ±2.8 | *34* | 38.6 | ±3.1 |
|  | Optimum 2.0 | *66* | 38.2 | ±2.9 | *34* | 38.5 | ±3.1 | *31* | 38.6 | ±2.4 | *20* | 40.1 | ±3.4 |
|  | sMVS | *68* | 37.8 | ±2.6 | *89* | 38.3 | ±2.9 | *28* | 39.2 | ±3.1 | *35* | 39.7 | ±3.1 |
|  | Non-users | *-* |  |  | *23* | 38.8 | ±2.7 | *26* | 39.8 | ±3.3 | *32* | 38.8 | ±3.1 |

Data are presented as mean ± standard deviation.

*MVS* multivitamin supplement*, sMVS* standard multivitamin supplement*, MCV* mean corpuscular volume*, PTH* parathyroid hormone.

^1^ Corrected for albumin levels (total calcium – (0.025 x albumin) + 1).

**
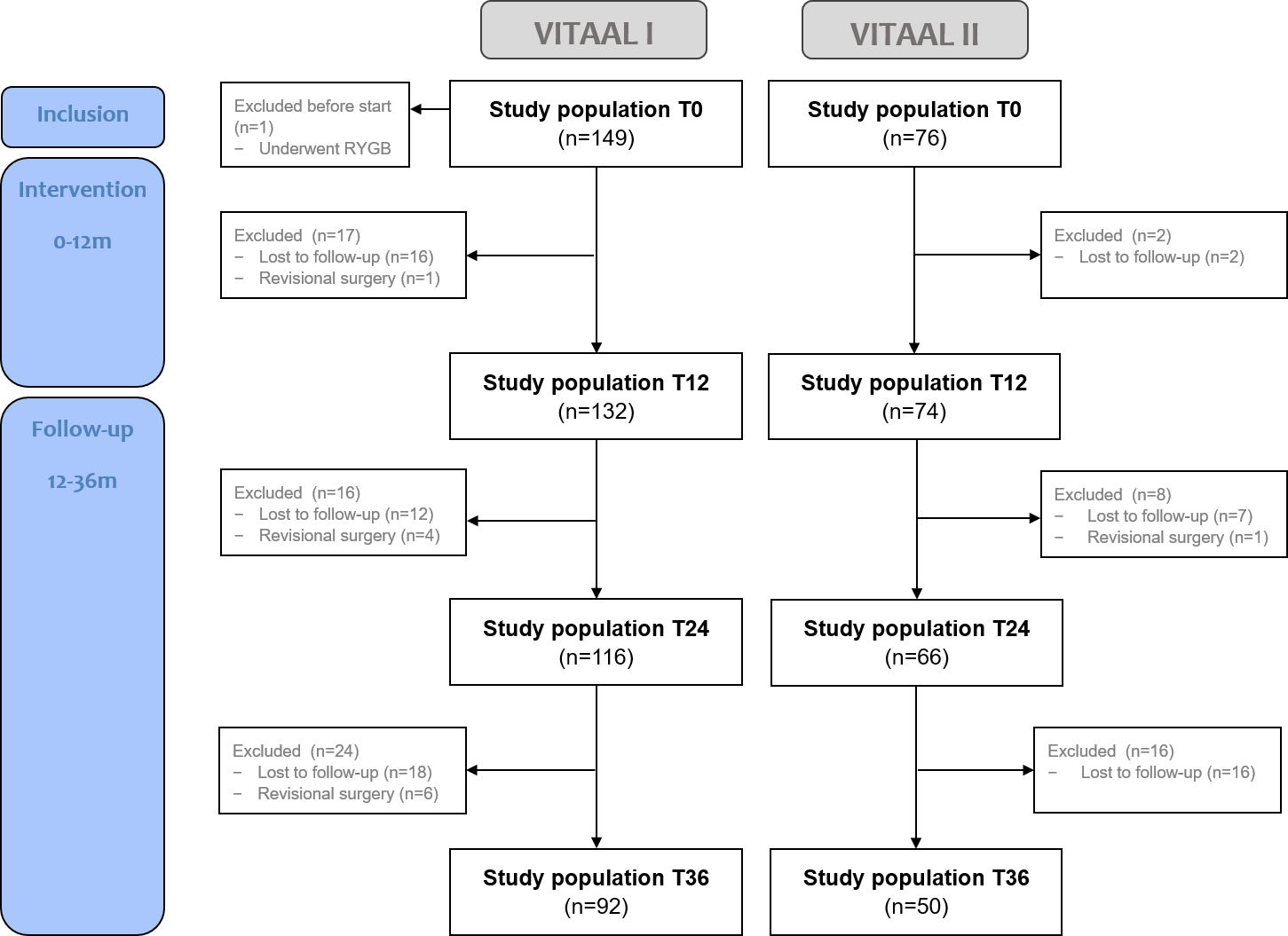
**

**Supplementary Figure 1.** Flowchart of the study population of the VITAAL I and VITAAL II study at baseline (T0), after the 12-month intervention period (T12), and at 24 and 36 months of follow-up (T24, T36).
